# Recent advances in photoredox and nickel dual-catalyzed cascade reactions: pushing the boundaries of complexity

**DOI:** 10.1039/d0sc00712a

**Published:** 2020-04-01

**Authors:** Chen Zhu, Huifeng Yue, Lingling Chu, Magnus Rueping

**Affiliations:** a KAUST Catalysis Center , KCC , King Abdullah University of Science and Technology , KAUST , Thuwal 23955-6900 , Saudi Arabia . Email: magnus.rueping@kaust.edu.sa; b State Key Laboratory for Modification of Chemical Fibers and Polymer Materials , Center for Advanced Low-Dimension Materials , College of Chemistry , Chemical Engineering and Biotechnology , Donghua University , Shanghai 201620 , China . Email: lingling.chu1@dhu.edu.cn

## Abstract

Cascade reactions that produce multiple chemical bonds in one synthetic operation are important in the efficient construction of complex molecules. In addition, photoredox and nickel dual catalysis opens a new and powerful avenue for transition-metal-catalyzed cross-coupling reactions. By combining these two concepts, photoredox and nickel dual-catalyzed cascade reactions have been recently established, and they provide an efficient and mild method for accessing a series of valuable organic compounds.

## Abstract 2

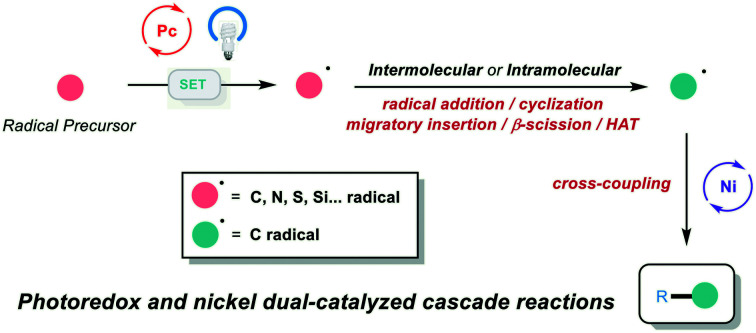
Cascade reactions that produce multiple chemical bonds in one synthetic operation are important in the efficient construction of complex molecules.

## 1. Introduction

**Scheme 1:**
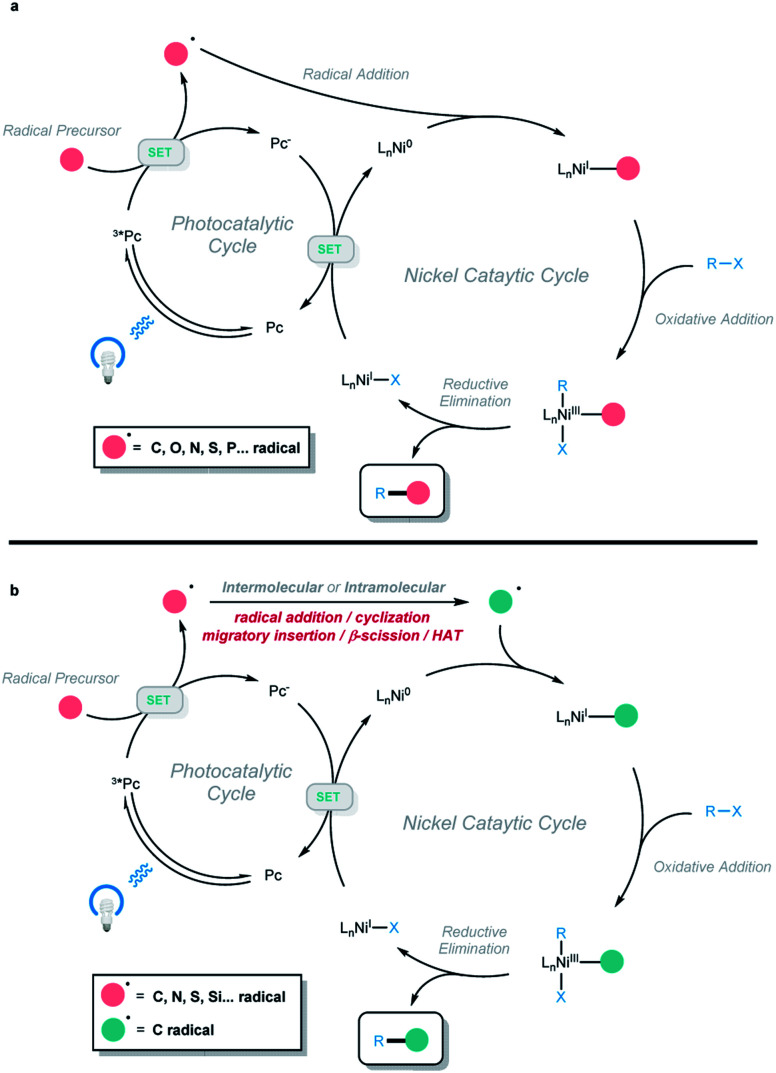
(a) Conventional photoredox and nickel dual-catalyzed two-component cross-coupling reactions. (b) Photoredox and nickel dual-catalyzed cascade cross-coupling reactions.

In recent years, photoredox and transition-metal dual catalysis has come to the fore in realizing a wide range of useful transformations for accessing valuable organic compounds.^1^,^2^ In particular, photoredox and nickel dual catalysis proved attractive due to the availability of various oxidation states of nickel catalysts, including Ni^0/I/II/III^.^3^,^4^ In this context, different types of radicals are generated (C, O, N, S, P, … radicals) *via* a single-electron-transfer event with the excited photocatalyst. Then, the radical is captured by a nickel complex, followed by the oxidative addition of aryl/alkyl/vinyl halides. The formed unstable Ni^III^ intermediate is prone to undergo reductive elimination, and the two-component cross-coupling product is obtained (Scheme 1a). Using this platform, different types of C–C and C–heteroatom bond-forming reactions have been developed, and excellent reviews covering this class of two-component cross-coupling reactions have been published.^3^ Very recently, a series of elegant photoredox and nickel dual-catalyzed cascade reactions were developed by our group and other groups, providing efficient and mild methods to access a series of valuable organic compounds from simple and abundant starting materials. In this process, the generated radical is not directly cross-coupled with an electrophile, but instead undergoes a cyclization, radical addition, migratory insertion, β-scission, or 1,5-HAT process to generate a new radical species that participates in the subsequent nickel-catalyzed cross-coupling to afford the final cascade product (Scheme 1b). This minireview focuses on the recent progress in photoredox and nickel dual-catalyzed cascade reactions according to the type of radical generated (with two non-radical exceptions).

We believe that this review will help researchers systematically understand this type of reactions, facilitating the further development of efficient cascade reactions under mild conditions.

## 2. C-radicals involved photoredox and nickel dual-catalyzed cascade reactions

Alkyltrifluoroborates,4m,^5^ alkylsilicates,4l,^6^ carboxylic acids,4n,^7^ alkyl oxalates,^8^ 4-alkyl-1,4-dihydropyridines (DHPs),4e,^9^ and THF^10^ have been demonstrated as readily available carbon radical precursors in photochemistry. Using photoredox and nickel dual-catalyzed cascade reactions as a platform, a series of protocols for the difunctionalization of alkynes and alkenes have been established using these radical precursors.

**Scheme 2:**
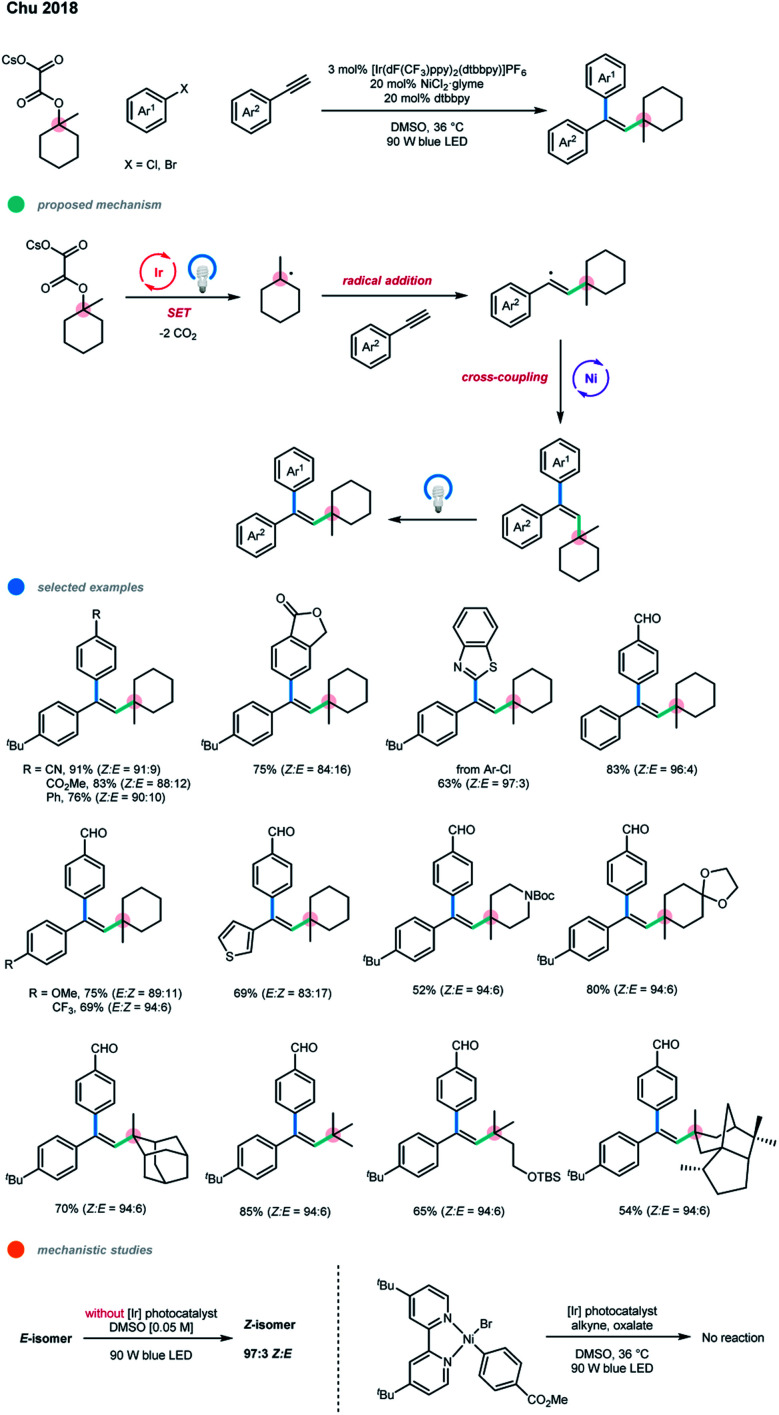
*Syn*-selective alkylarylation of terminal alkynes *via* photoredox and nickel dual catalysis.

In 2018, Chu and coworkers developed an intermolecular three-component cascade reaction for the first time, realizing the regioselective, *syn*-alkylarylation of terminal alkynes *via* photoredox and nickel dual-catalyzed cross-coupling reaction of tertiary alcohol derivatives with alkynes and aryl bromides (Scheme 2).^11^ Mechanistically, a tertiary radical is generated upon single-electron oxidation of a tertiary alkyl oxalate by an excited Ir photocatalyst with the loss of two molecules of CO_2_. The resulting alkyl radical adds regioselectively to a terminal alkyne to give a linear alkenyl radical, and subsequent *anti*-addition to Ni^0^ gives an *E*-alkenyl–Ni^I^ complex. The *E*-alkenyl–Ni^I^ complex undergoes oxidative addition with aryl bromide and reductive elimination to afford *E*-alkene product. Finally, *E* → *Z* isomerization of the *E*-product *via* a photoinduced energy transfer pathway produces the *Z*-isomer of the product. Notably, an experimental mechanistic investigation showed that the photocatalyst does not engage in the isomerization step as the formed trisubstituted *E*-alkene itself could act as a photosensitizer for the isomerization process. In addition, the aryl–Ni^II^–Br complex was prepared, and its reaction with the alkyne and oxalate gave no desired cascade product, supporting the proposed Ni^0/I/III^ catalytic pathway.

A series of electron-deficient aryl bromides and benzothiazole-derived heteroaromatic chlorides participate smoothly in this reaction, giving the corresponding products in good to high yields with excellent stereoselectivities. Terminal alkynes bearing electron-neutral, electron-donating, or electron-withdrawing phenyl motifs or thiophene could all undergo this transformation to form the desired products with high to excellent yields and stereoselectivities. In addition, a wide range of tertiary alkyl oxalates, including heterocyclic derivatives, could be used to synthesize the corresponding trisubstituted alkenes. DFT-based conformational analysis indicated that the photoinduced *E* → *Z* isomerization may result from the deconjugation of the π-system in the *Z*-isomers. The only shortcoming of the current methodology is the application of electron-rich aryl halides and primary and secondary carbon radical precursors.

**Scheme 3:**
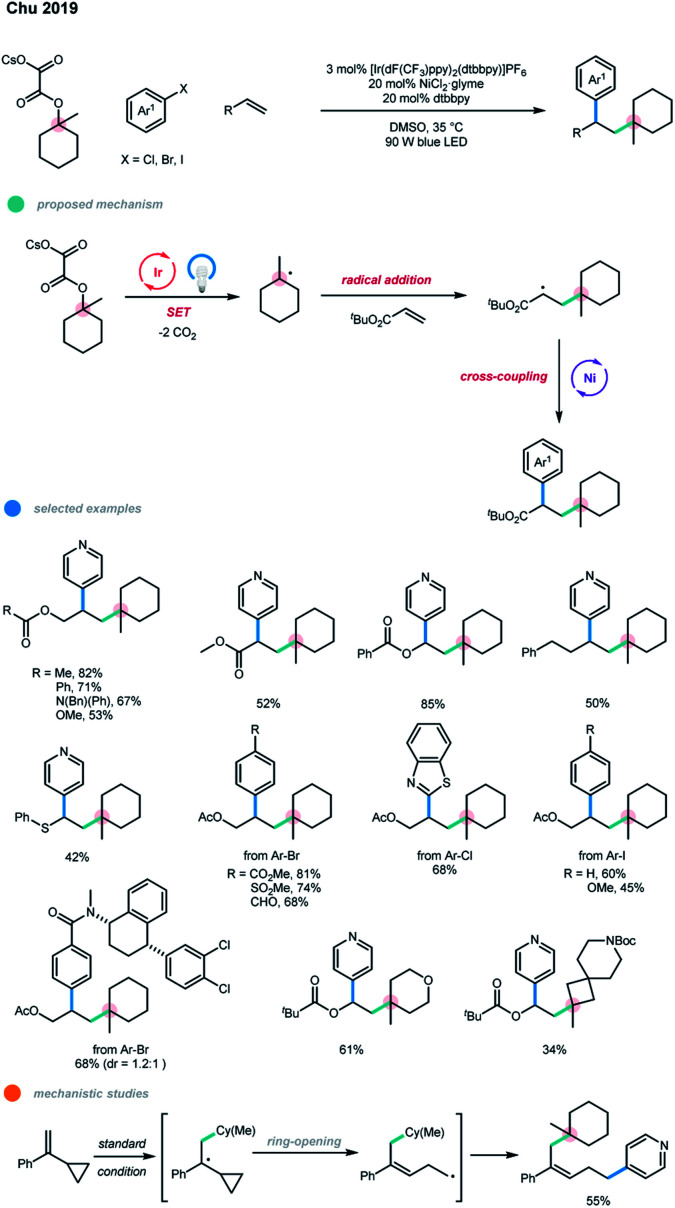
Intermolecular alkylarylation of alkenes *via* photoredox and nickel dual catalysis.

A short time later, the same group extended this photoredox and nickel dual-catalyzed cascade system to the intermolecular alkylarylation of alkenes (Scheme 3).^12^ A radical clock experiment was conducted with α-cyclopropyl-substituted styrene and the rearranged cascade product was generated in moderate yield, suggesting that a radical relay process is involved. Unactivated alkenes bearing esters, carbamates, and carbonates, as well as 4-phenyl-1-butene, all smoothly underwent the cascade difunctionalization with high efficiency. An array of electron-deficient and electron-rich alkenes were all viable substrates for this reaction. The scope with respect to aryl halides and alkyl oxalates is broad. Although electron-rich and electron-neutral aryl bromides seemed inefficient in this system, electron-rich and electron-neutral aryl iodides reacted efficiently. Notably, a number of complex molecules were also suitable for this protocol. It should be pointed out that the experimental results show that six-membered oxalates gave higher yields compared to four-, five-, and seven-membered systems.

**Scheme 4:**
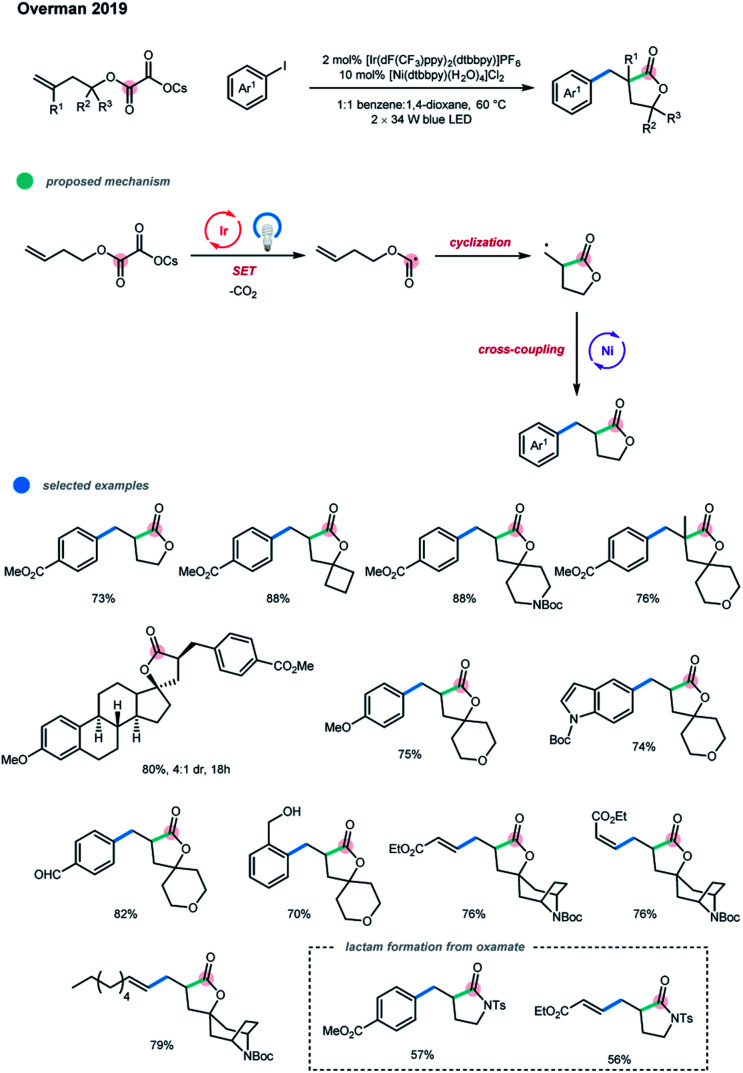
Preparation of spirolactones by an alkoxycarbonyl radical cyclization-cross-coupling cascade.

In addition to these two elegant three-component intermolecular cascades, the intramolecular cyclization-cross-coupling cascade of homoallylic cesium oxalates with aryl- and vinyl-iodides was reported by Overman and coworkers *via* photoredox and nickel dual catalysis in the same year (Scheme 4).^13^ The facile construction of pharmaceutically relevant spirolactones was realized under mild conditions. The key factor in the success of this cascade transformation is that the 5-*exo* cyclization of the alkoxycarbonyl radical occurs faster than the decarboxylation. The carbon-centered radicals generated upon cyclization could be intercepted by nickel and undergo further cross-coupling with aryl iodides. Aryl bromides could also be employed, albeit with lower efficiency. The utilization of cesium as the counterion of the oxalate substrate is crucial for the productivity of the reaction as no desired product was observed when the corresponding lithium salt was used. A series of homoallylic cesium oxalates containing geminal dimethyl moieties, different size rings, oxygen/nitrogen heterocycles, adamantane, and even the natural product scaffold estrone all participated well in this cascade, furnishing the corresponding lactones in good yields. Notably, challenging quaternary carbon center can be created in this cyclization/cross-coupling cascade. In addition, an array of aryl iodides, especially those bearing sensitive functionalities such as unprotected benzylic alcohols and free aldehydes, could also be applied. Both *E-* and *Z*-vinyl iodides, including unactivated ones, undergo this coupling with complete stereoretention. Moreover, functionalized lactam products could be obtained in good yields *via* the cascade reaction of the oxamate substrate with aryl and vinyl iodides.

**Scheme 5:**
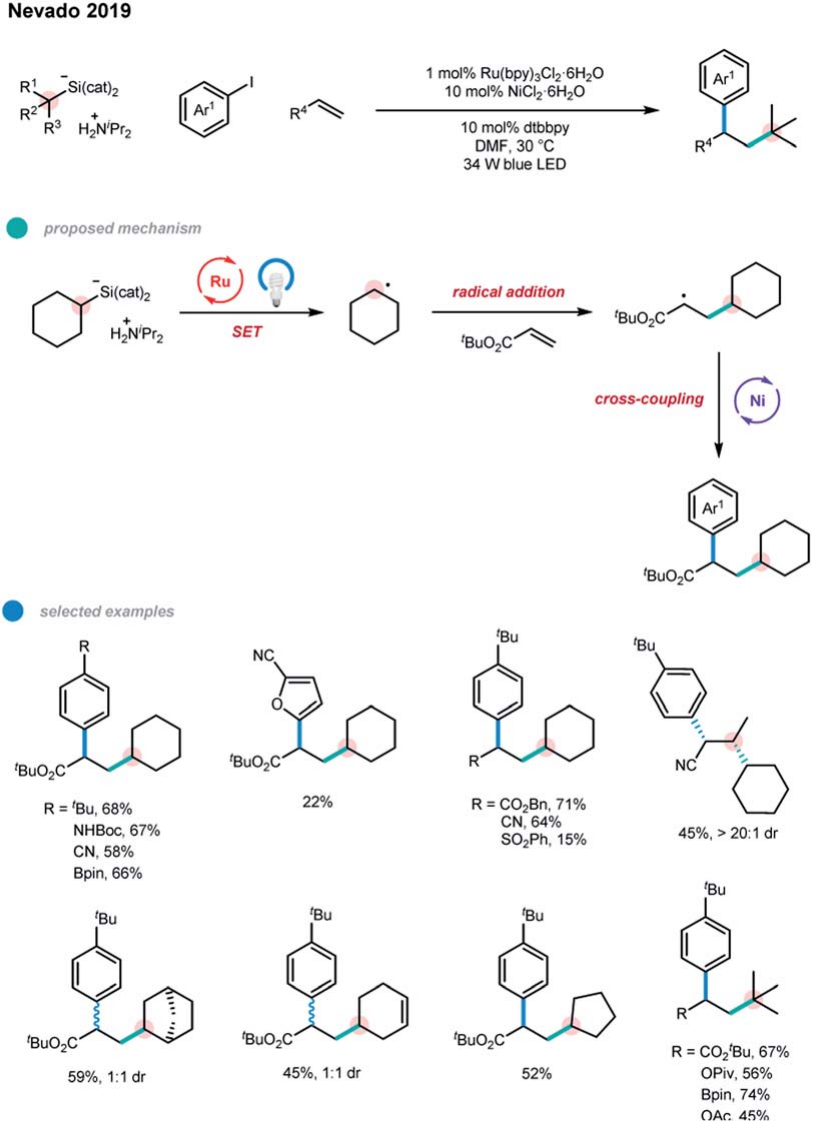
Three-component alkylarylation of alkenes *via* photoredox and nickel dual catalysis.

Alkyl silicates were successfully employed in the photoredox and nickel dual-catalyzed cascade system by Nevado and coworkers to achieve the arylalkylation of alkenes under mild conditions (Scheme 5).^14^ For this process, the alkyl silicate first undergoes a SET event with an excited Ru photocatalyst to give an alkyl radical. The radical then adds to a double bond to generate a new alkyl radical that can then participate in the subsequent nickel-catalyzed cross-coupling with an aryl iodide. The molar ratio between the three coupling partners is crucial for the formation of the arylalkylation products. Slight excesses of alkene and alkyl silicate help to produce the three-component products while minimizing the two-component coupling between alkyl silicate and aryl iodide. A series of aryl iodides as well as secondary and tertiary silicates could be used in this protocol with good efficiency. In addition to activated alkenes bearing electron-withdrawing groups, electron-rich olefins, vinylboranes, and even unactivated alkenes could also be coupled with tertiary silicates. Hiyama-type coupling products between alkyl silicates and aryl iodides can be completely avoided by using tertiary alkyl silicates.

**Scheme 6:**
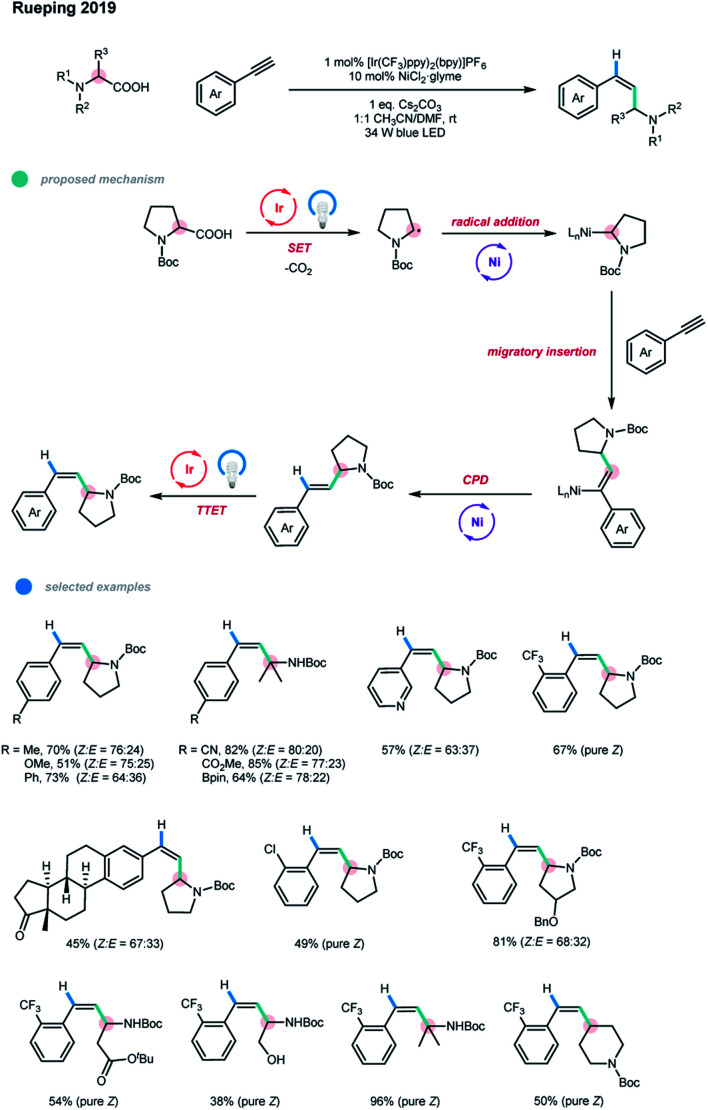
Hydroalkylation of alkynes *via* photoredox and nickel dual catalysis.

Very recently, Rueping and coworkers developed a photoredox and nickel dual-catalyzed protocol for the *anti*-Markovnikov hydroalkylation of terminal alkynes and a one-pot alkylarylation of alkynes (Scheme 6).^15^ A series of valuable disubstituted and more challenging trisubstituted alkenes could be obtained in a *syn*-addition manner under mild conditions. The mechanism starts with the oxidation of the feedstock starting material, an alkyl carboxylic acid, with an excited Ir photocatalyst. The loss of one molecule of CO_2_ generates an alkyl radical. Subsequent radical trapping with Ni species and SET reduction give an alkyl–Ni^I^ complex, which then undergoes an alkyne migratory insertion to deliver a *syn*-alkenyl–Ni^I^ complex. For hydroalkylation, concerted protonation demetallation (CPD) of the *syn*-alkenyl–Ni^I^ complex gives the *E* isomer of the hydroalkylation product, followed by an energy transfer step with an excited photocatalyst to finally form a *Z*-isomer of the product. For arylalkylation, the *syn*-alkenyl–Ni^I^ complex undergoes Ni-assisted isomerization, oxidative addition of the aryl halide, and reductive elimination to produce the *anti*-addition cascade coupling product. Likewise, the *syn*-addition product is obtained *via* a subsequent energy transfer pathway. The steady-state and time-resolved fluorescence quenching study confirmed the irreversible SET process between the photocatalyst and deprotonated carboxylic acid. DFT calculations also supported the proposed mechanism. Unlike previous reports, the hydroalkylation reaction proceeds without an external reductant, such as a hydrosilane or protic solvent. Moreover, no ligand is needed for this process.

The scope of the alkyne hydroalkylation with respect to both reaction partners is quite broad. A series of terminal alkynes bearing diverse electron-rich and electron-deficient aryl groups could undergo this reaction with moderate to high efficiency. Borate ester, chloride and even bromide groups on the aryl alkyne were well tolerated. Notably, ethynylbenzenes containing *ortho*-chloride and -trifluoromethyl functional groups could produce exclusively the *Z* isomers of the corresponding products, and the reaction can be scaled up without decreases in yield or stereoselectivity. A wide range of Boc-protected cyclic and acyclic secondary amino acids could be reacted with 1-ethynyl-2-(trifluoromethyl)benzene in moderate to high yields with excellent stereoselectivity. Notably, Boc-protected tertiary amino acids offered significantly improved reaction efficiencies.

**Scheme 7:**
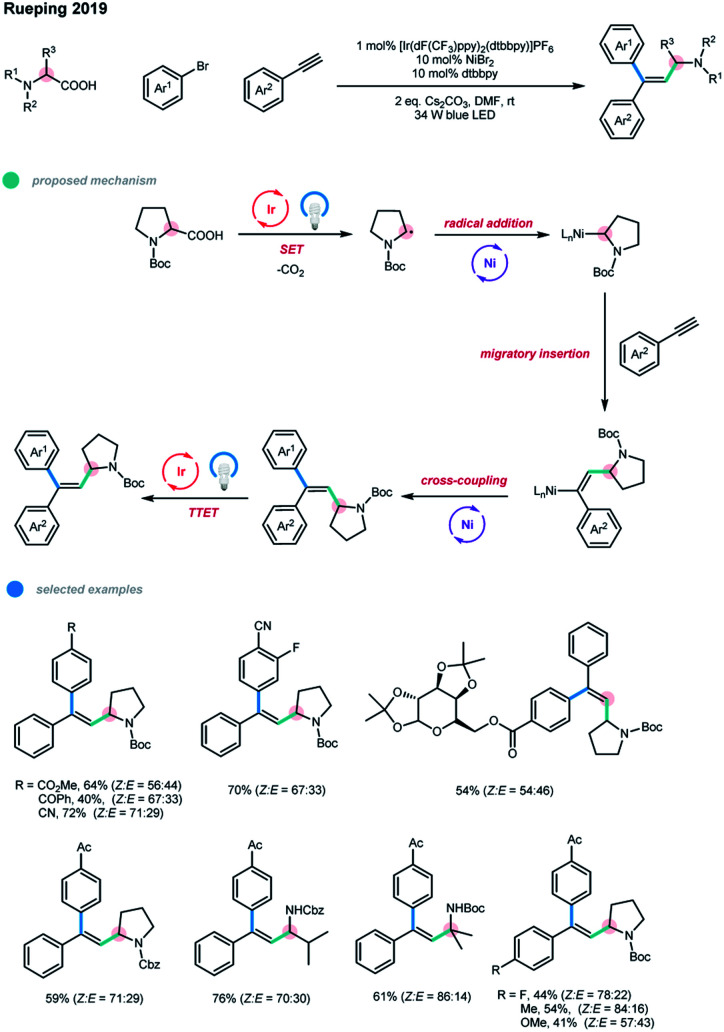
Arylalkylation of alkynes *via* photoredox and nickel dual catalysis.

After a minor modification of the reaction conditions, the authors also realized a three-component cascade coupling reaction of alkynes with carboxylic acids and aryl bromides (Scheme 7).^15^ Using this methodology, diverse trisubstituted alkenes were obtained in moderate to good yields with the *syn*-addition products being favored. Moreover, pharmaceutically relevant heterocyclic and natural product-derived aryl bromides were also well suited for this reaction.

**Scheme 8:**
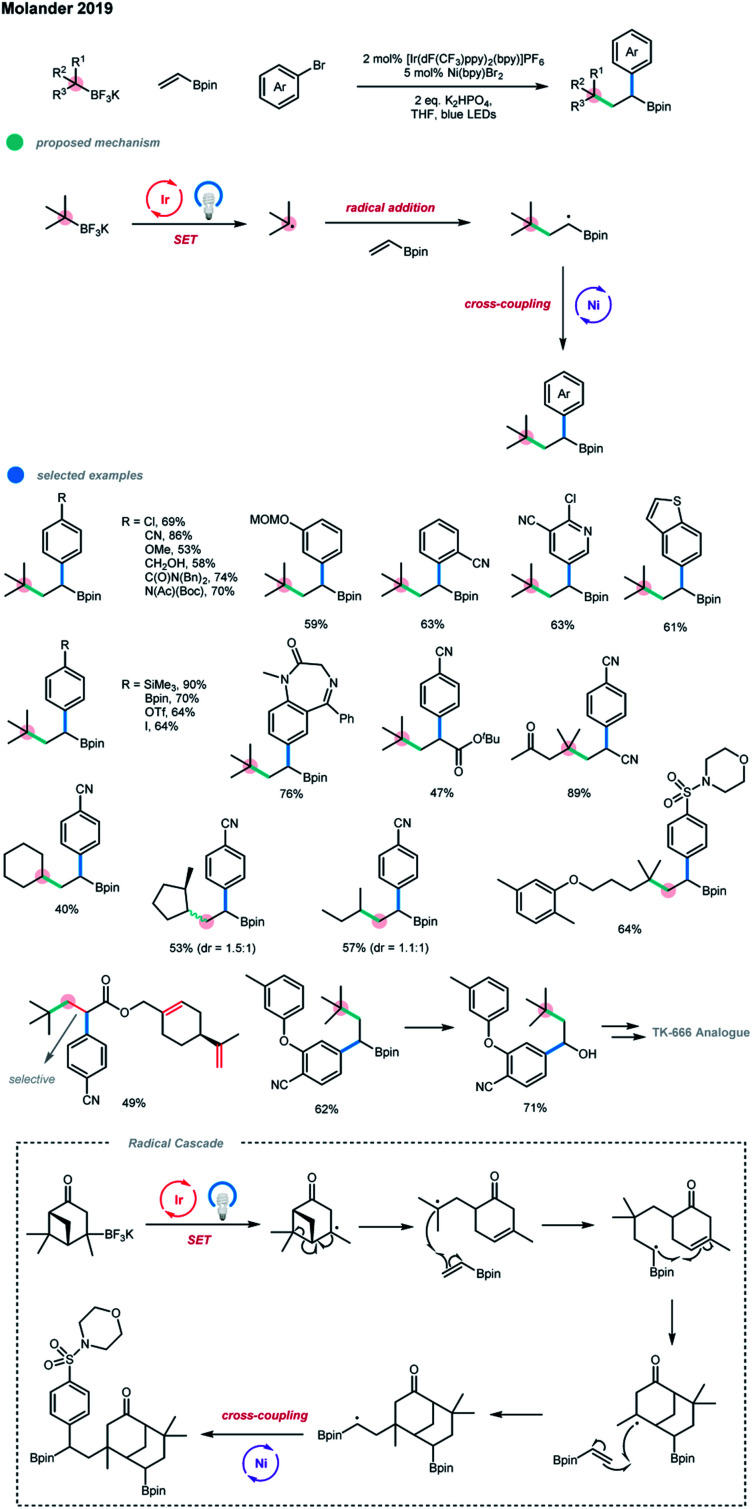
Olefin dicarbofunctionalization *via* photoredox and nickel dual catalysis.

A photoredox and nickel dual-catalyzed dicarbo-functionalization of olefins *via* intermolecular three-component coupling of alkyltrifluoroborates, vinyl boronate, and aryl bromides was recently developed by Molander and coworkers (Scheme 8).^16^ Both quaternary and tertiary carbon centers could be formed in a single step. Moreover, pendent pinacol boronate esters on the generated products allow further derivatization and late-stage functionalization. Mechanistically, the carbon radical generated from a SET oxidation by the excited state of the photocatalyst first undergoes a Giese-type addition to an olefin, generating a secondary carbon radical bearing Bpin group, and this species subsequently undergoes a nickel-catalyzed coupling with an aryl halide.

The success of this three-component dicarbofunctionalization depends on the utilization of radicals resistant to direct coupling with aryl halides. Electron-poor, electron-neutral and electron-rich aryl bromides, as well as pharmaceutically relevant heterocyclic bromides, were all suitable for this protocol, demonstrating a wide functional group tolerance. For some substrates, the protodeborylation products were obtained, which may be due to the combined effect of the Lewis basicity of the N-containing heterocycle and the α-substituted electron-withdrawing groups. Interestingly, the monofunctionalization of a diiodide was achieved, leaving one iodide group for further cross-coupling. Although tertiary and secondary alkyltrifluoroborates could both be employed in this three-component coupling system, tertiary alkyltrifluoroborates have advantages as the olefin difunctionalization is 16 times faster than the two-component coupling reaction with an aryl bromide for tertiary radicals, but it is only 2.5 times faster for secondary radicals. Notably, verbenone-derived trifluoroborate gave an unexpected bisborylated product *via* a unique radical cascade pathway. When substrates bearing different types of olefins were employed, 1,2-difunctionalization selectively occurred on the electron-deficient olefin. In addition, a series of the alkylarylated products bearing pinacolboronate esters were subjected to further derivatization reactions, and diverse and valuable compounds, including TK-666 intermediate analogues, were obtained. The authors also pointed out that substrates with more stable benzyl, α-oxy and α-amino groups failed to give the desired products due to their low reactivity or the competitive two-component alkylation reaction of aryl bromides.

**Scheme 9:**
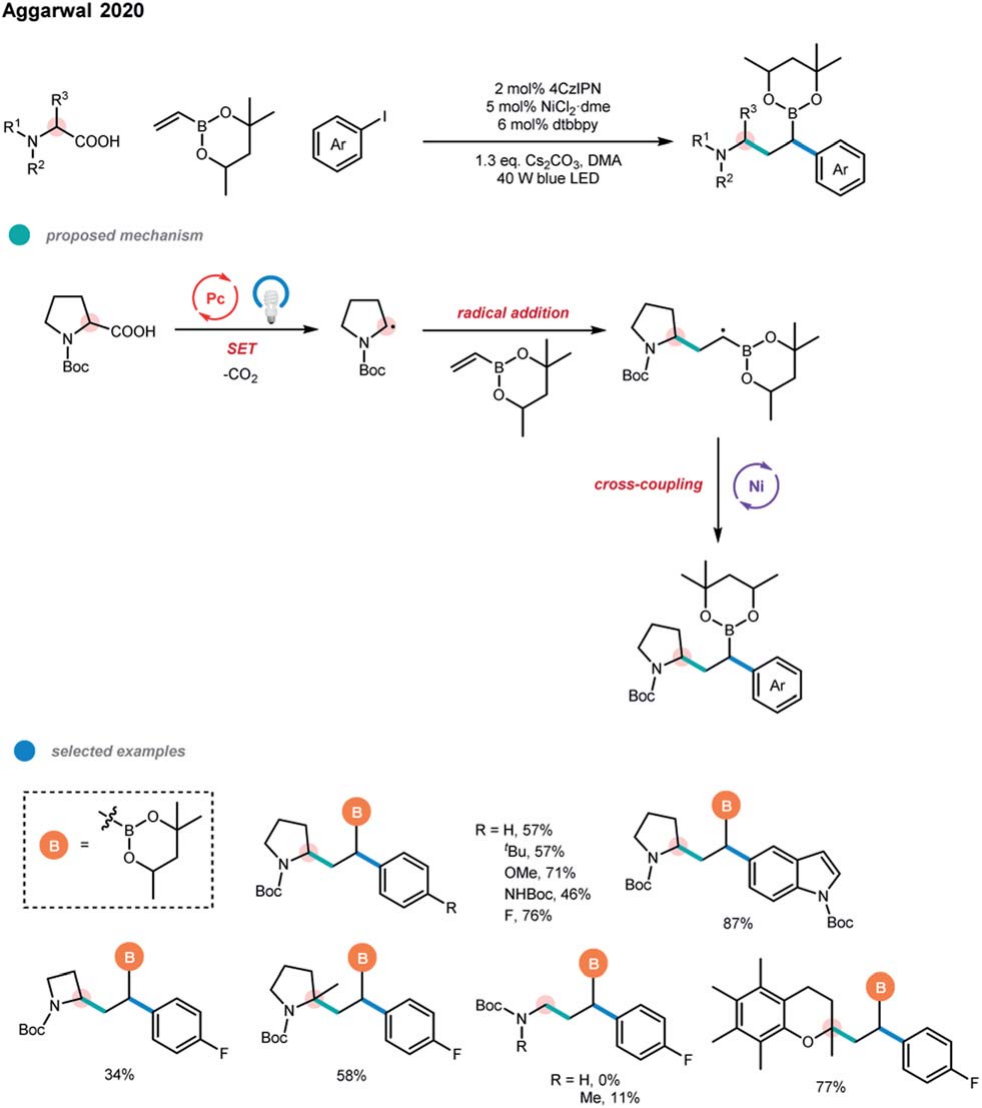
Decarboxylative conjunctive cross-coupling of vinyl boronic esters *via* photoredox and nickel dual catalysis.

Carboxylic acids were also successfully applied in the dicarbofunctionaliztion of vinyl boronate by Aggarwal and coworkers (Scheme 9).^17^ A wide range of complex alkyl boronic esters was synthesized *via* the photoredox and nickel dual-catalyzed three-component decarboxylative cascade cross-coupling reaction under mild conditions. The excellent regioselectivity of this cascade transformation relies on the formation of a stabilized α-boryl radical, in which the unpaired electron is delocalized in the empty π-orbital of the adjacent boron atom. Primary, secondary and tertiary α-amino acids and several biologically relevant α-oxy acids were suitable substrates. In addition, a series of electron-rich aryl and heterocyclic iodides could be employed to give the cascade products in good yields.

Moreover, several *sedum* alkaloids were synthesized using this method, highlighting the practicability of the procedure. However, electron-deficient aryl iodides failed to give the desired boronic ester products that are prone to undergo protodeboronation, and aryl bromides were not suitable for this three-component cascade reaction due to the competitive formation of two-component Giese products between carboxylic acids and vinyl boronates.

**Scheme 10:**
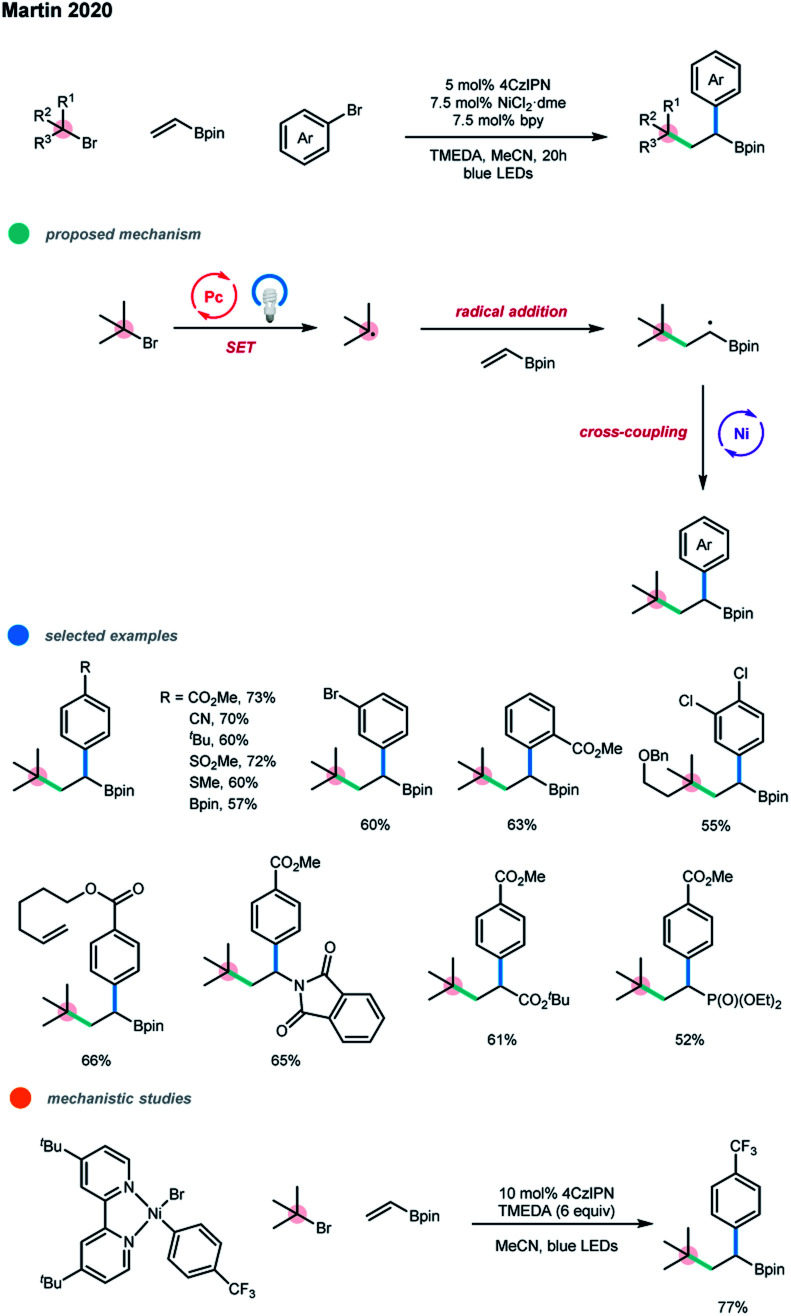
Dicarbofunctionalization of vinyl boronates *via* photoredox and nickel dual catalysis.

Simultaneously, Martin and coworkers realized the 1,2-dicarbofunctionaliztion of vinyl boronates *via* the photoredox and nickel dual-catalyzed cascade cross-electrophile coupling of aryl bromides, vinyl boronates and tertiary alkyl bromides (Scheme 10).^18^ This transformation provided the desired alkyl boronates in high chemo- and regioselectivity. Notably, the two-component cross-coupling products between any two coupling partners were suppressed effectively. The radical was generated by a SET reduction by the excited photocatalyst and subsequently added regioselectively to the vinyl boronate to give a stabilized α-boryl radical. This α-boryl radical participated in the nickel catalyzed cross-electrophile coupling with an aryl bromide, delivering the three-component cascade product. The photocatalysis cycle completed with TMEDA as a sacrificial donor. The aryl–Ni^II^–Br complex prepared from 4-trifluoromethyl bromobenzene and Ni(cod)_2_ could give the desired cascade product in good yield only with the assistance of 6 equiv. TMEDA, supporting the proposed Ni^II/III/I^ reductive cross-coupling mechanism. The electronic and steric properties of aryl bromides had no significant influence on the yield of cascade products and a wide range of electron-donating and electron-withdrawing functional groups were well tolerated. It should be pointed out that LiCl was used as an additive for some substrates in order to obtain high yields. A number of tertiary alkyl bromides and several olefin acceptors other than vinyl boronate were tested and all gave the corresponding cascade coupling products. However, primary and secondary alkyl bromides seem ineffective in this protocol.

## *3*. N-radical-involved photoredox and nickel dual-catalyzed cascade reactions

Amidyl radical generation from non-prefunctionalized N–H bonds has been seen as a significant challenge for a long time due to the high stability of these bonds.^19^ A major advance was made by the Knowles group and Rovis group, who took advantage of proton-coupled electron transfer (PCET) and photoredox catalysis to enable the formation of the amidyl radicals from non-prefunctionalized N–H bonds under mild conditions.^20^

**Scheme 11:**
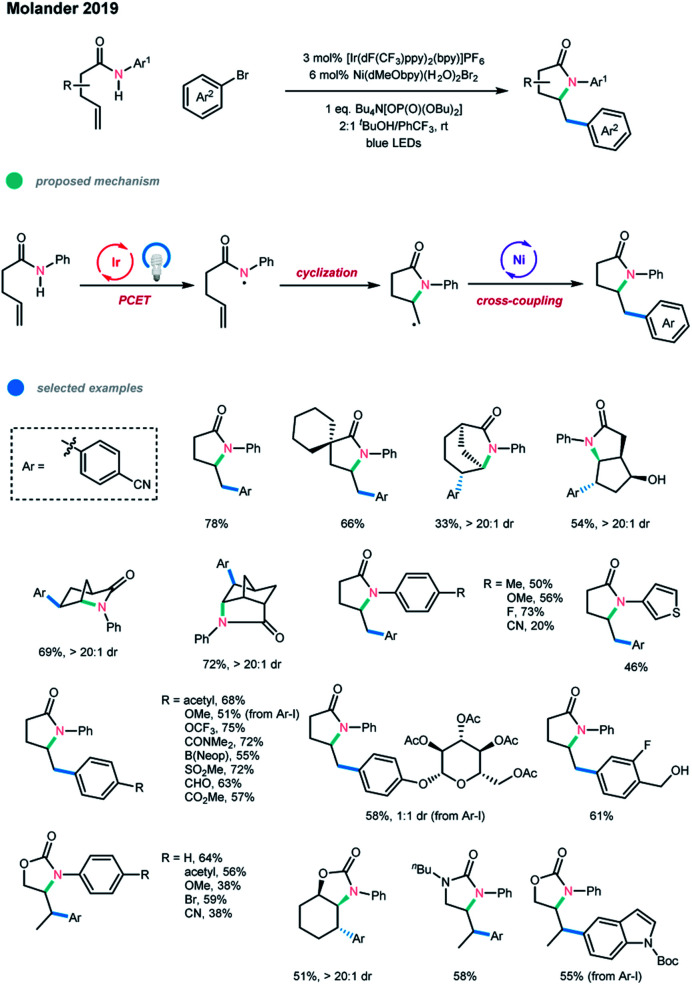
Cascade amidoarylation of unactivated olefins *via* merging photoredox PCET with nickel catalysis.

Very recently, Molander and coworkers elegantly combined photoredox PCET and nickel catalysis for the first time, realizing the cascade amidoarylation of unactivated olefins in a highly diastereoselective manner (Scheme 11).^21^ In this process, an amidyl radical was first generated *via* a concerted PCET and photocatalyzed pathway, followed by a fast 5-*exo*-trig cyclization. The resultant alkyl radical was then involved in a nickel-catalyzed cross-coupling reaction with an aryl halide to give a 5-membered N-containing heterocyclic product. In contrast to the previous reports by Knowles, a larger amount of a phosphate base (Bu_4_N[OP(O)(OBu)_2_] (2.5 equiv.)) was necessary to achieve a high cross-coupling yield. A 2 : 1 mixture of ^*t*^BuOH/PhCF_3_ was chosen as the optimal reaction solvent, most likely because ^*t*^BuOH can enhance the acidity of the N–H bond, thus facilitating the formation of the amidyl radical, and PhCF_3_ has beneficial effects in radical reactions. The transformation occurred with high efficiency for electron-neutral and electron-rich amides. However, electron-deficient amides gave the corresponding products in lower yields, which may be attributed to their higher oxidation potentials. The scope with regard to aryl halides is broad, as illustrated by the tolerance of a series of functional groups, including activated benzylic alcohols and aldehydes, and by the application of heteroaryl halides. Notably, carbamates and ureas bearing terminal or internal alkenes were also suitable substrates for this cascade cross-coupling system.

**Scheme 12:**
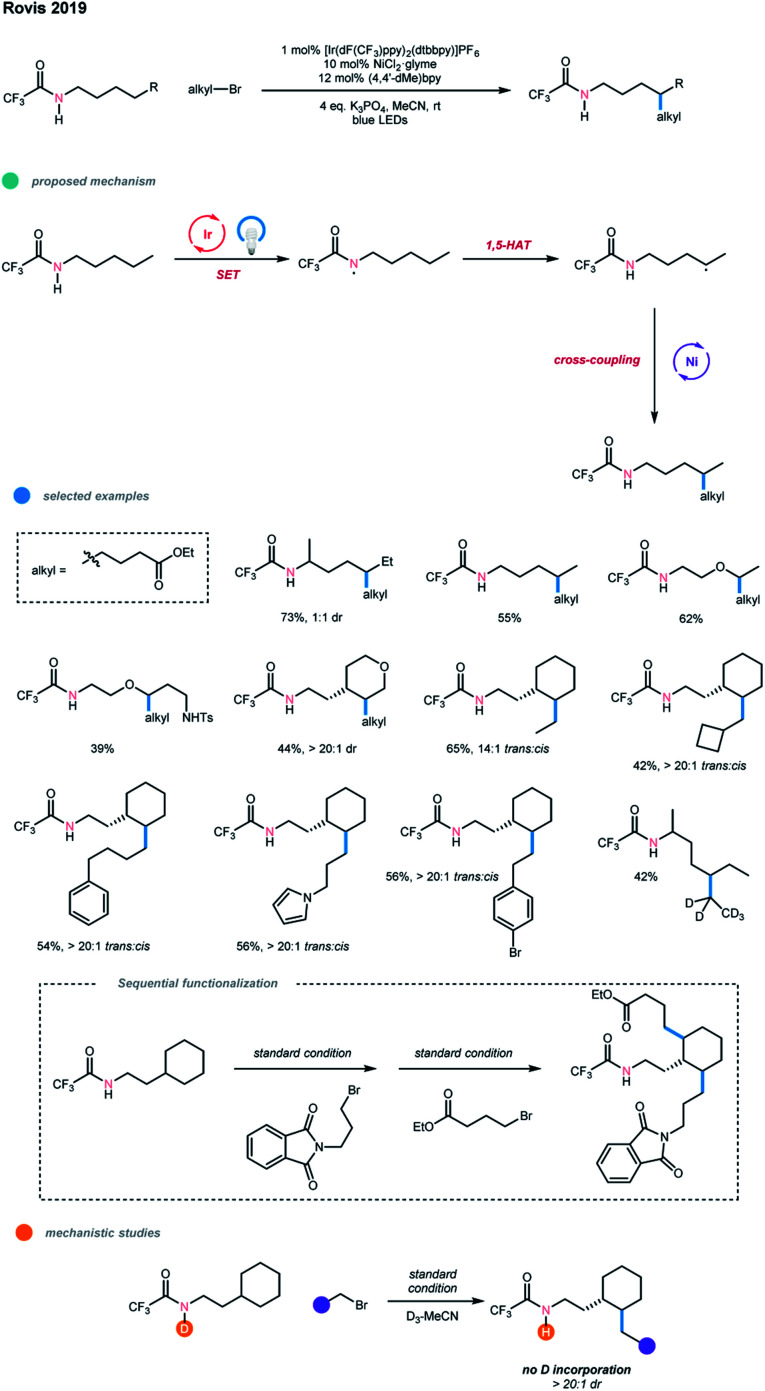
Regioselective alkylative cross-coupling of remote unactivated C(sp^3^)–H bonds *via* photoredox and nickel dual catalysis.

In the same year, using trifluoroacetamides bearing unactivated C(sp^3^)–H bonds, Rovis and coworkers realized the alkylation of distal unactivated C(sp^3^)–H bonds with high regioselectivity through a cross-coupling with alkyl bromides *via* a photoredox and nickel dual-catalyzed cascade (Scheme 12).^22^ Mechanistically, the acidic trifluoroacetamide directing group is first deprotonated by K_3_PO_4_ as a base and oxidized by the excited-state photocatalyst to deliver a neutral amidyl radical. This amidyl radical then undergoes an intramolecular 1,5-HAT to generate a carbon-centered radical at the δ-position, which can be captured by a nickel catalyst and undergo cross-coupling with an alkyl bromide. Deuterium labeling of the amidyl N–H gave no deuterium containing product, demonstrating the intramolecular 1,5-HAT process is involved in the catalytic cycle.

Notably, the directing trifluoroacetamide is essential for the success of this process; other directing groups, such as phenylacetamide, show no reactivity due to the decrease in the acidity of the N–H proton. Both aliphatic linear and cyclic amides work well in this reaction, and the *trans*-products were obtained with high diastereoselectivity from cyclic substrates. Importantly, the reaction can selectively functionalize the secondary δ-C(sp^3^)–H bonds of amides and give the monoalkylated products at the δ-methylene site even when the substrate amide contains additional activated C(sp^3^)–H bonds at other positions. A wide range of primary alkyl bromides bearing diverse functional groups, including protected alcohol, nitrile, alkene, dioxolane, phthalimide, pyrrole, and aryl bromide, could all smoothly undergo this cascade reaction. Difunctionalization with two different alkyl groups could also be realized when the amide has additional δ-methylene sites. A limitation of this methodology is that it seems not tolerant of other alkyl electrophiles, such as secondary and tertiary alkyl bromides, benzylic bromides, alkyl chlorides, alkyl iodides, tosylates and triflates.

**Scheme 13:**
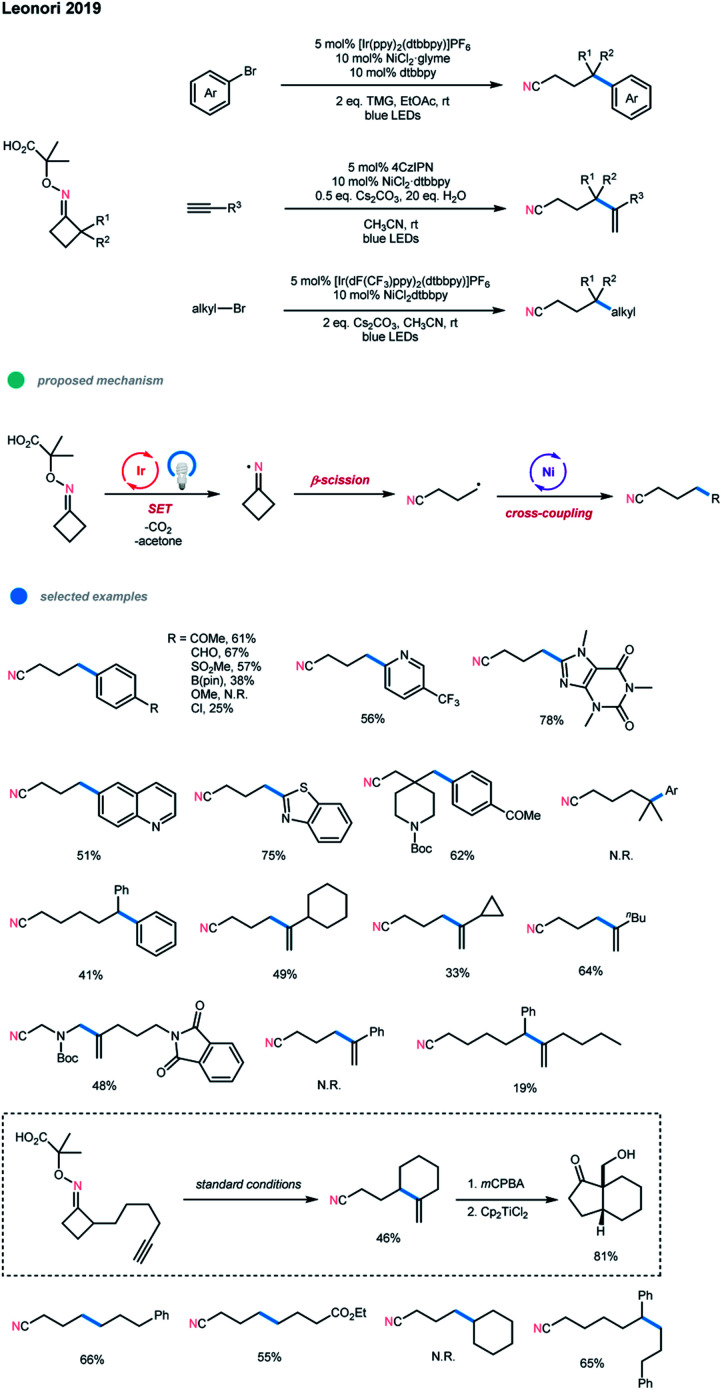
Radical ring-opening-arylation, -vinylation and -alkylation cascades *via* photoredox and nickel dual catalysis.

Another excellent example of the N-radical-involved cascade cross-coupling reaction was developed by Leonori and coworkers (Scheme 13).^23^ Using oxime-carboxylates as radical precursors, they synthesized a series of remotely functionalized nitriles *via* photoredox and nickel dual-catalyzed radical ring-opening-arylation, -vinylation and -alkylation cascades. The reaction began with photocatalyst excitation and SET oxidation of oxime-carboxylate. Subsequent extrusion of CO_2_ and acetone generates an iminyl radical, which then undergoes a ring-opening to deliver a distal nitrile radical. The resulting distal nitrile radical is then coupled with an aryl/alkyl halide or alkyne *via* nickel catalysis, affording a series of distal arylated, vinylated, and alkylated nitrile-containing products. For the arylation cascade, a wide range of electron-deficient aryl bromides and heteroaromatic bromides bearing differentially functionalized pyridines, quinolone, benzothiazole, benzimidazole, and caffeine could all be employed, while electron-rich aryl bromides failed to give the corresponding products.

Cyclobutanone and cyclohexanone oximes smoothly under-went this coupling reaction. gem-Diemthyl cyclopentanone oxime gave no desired product, which may be because the addition of tertiary C-radicals to the Ni center is difficult. Moreover, this arylation cascade has been achieved on a large scale using flow chemistry. For the vinylation cascade, several linear and cyclic alkyl-substituted terminal alkynes were shown to be suitable substrates. However, aryl-substituted alkynes and larger ring-size iminyl precursors gave no products or lower yields. Additionally, intramolecular radical ring-opening vinylation could take place to form *gem*-alkene products with larger rings in moderate yields. For the alkylation cascade, many primary alkyl bromides and cyclobutanone and 2-Ph-cyclohexanone oximes could react in moderate to good yields. The current limitation of this alkylation cascade mode is that it is not tolerant of secondary alkyl electrophiles.

## 4. S-radical-involved photoredox and nickel dual-catalyzed cascade reactions

**Scheme 14:**
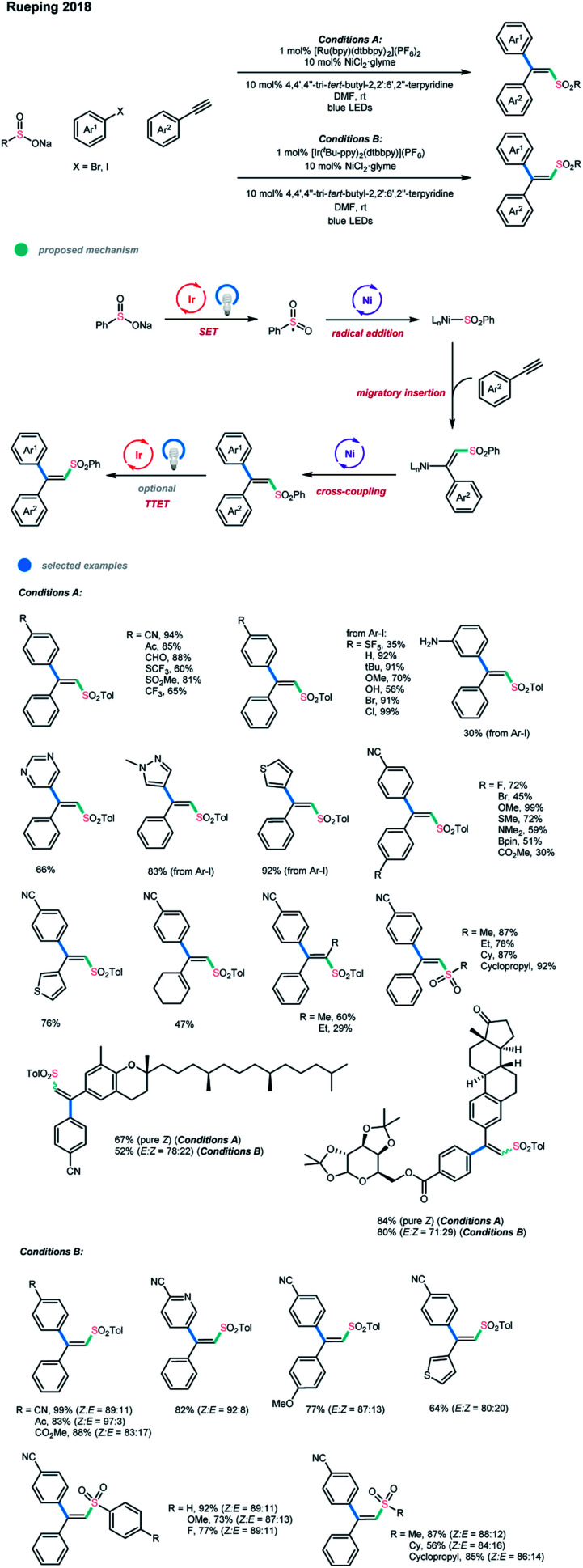
Three-component synthesis of stereodefined olefins *via* single-electron transfer, nickel catalysis, and triplet energy transfer.

In 2018, Rueping and coworkers reported the first efficient photoredox and nickel dual-catalyzed sulfonylation reaction of aryl, heteroaryl, and vinyl halides.4*o* One year later, the same group extended this system to a three-component cascade cross-coupling reaction to realize the difunctionalization of alkynes (Scheme 14).^24^ The arylsulfonylation of alkynes with high chemo-, regio-, and stereo-selectivity was achieved *via* the cross-coupling of aryl halides with sodium sulfinates and alkynes under mild conditions. Notably, the cross-coupling products of trisubstituted alkenes can be obtained with either *E*- or *Z*-selectivity by choosing an appropriate photocatalyst with a suitable triplet state energy. The results indicated that the reaction outcome is dependent on the photocatalyst, which promotes either a single-electron transfer process or both a single-electron transfer and an energy transfer process. The mechanism starts with a SET oxidation of the radical precursor (sodium sulfinate) by the excited photocatalyst to generate a sulfonyl radical.

The radical is then trapped by the Ni^I^ complex to give an alkyl–Ni^II^ complex, which is subsequently reduced by the photocatalyst and undergoes an alkyne migratory insertion and Ni-assisted *anti*/*syn* isomerization to deliver an alkenyl–Ni^I^ complex. The resulting alkenyl–Ni^I^ complex then undergoes an oxidative addition of an aryl halide and a reductive elimination to afford the three-component *anti*-addition product. At this stage, if the employed photocatalyst has a suitable triplet state energy, a photo-induced isomerization of the generated *anti*-addition alkene product would give the *syn*-addition product. When the Ru-based photocatalyst [Ru(bpy)(dtbbpy)_2_](PF_6_)_2_ was used, the *anti*-addition alkene products were obtained. A series of aryl bromides and aryl iodides bearing electron-withdrawing, electron-neutral and electron-donating substituents could be applied in this reaction with good to high efficiency. Additionally, heterocyclic substrates such as quinoline, pyridine, pyrimidine, pyrazole, and thiophene derivatives could all participate well in this reaction. In addition to aryl (and heteroaryl) alkynes bearing diverse functional groups, cyclohexenyl alkynes and internal alkynes were also suitable substrates for this cascade protocol. A series of structurally diverse sodium aryl sulfinates were shown to have high reactivities, and sodium alkyl sulfinates also gave the corresponding products in high yields. Remarkably, a series of complex molecules could react with high efficiency, showing the practicality of this novel cascade reaction and its great potential in late-stage synthesis.

When the Ir-based photocatalyst [Ir(^*t*^Bu-ppy)_2_(dtbbpy)](PF_6_) was used, *syn*-addition alkenes were obtained as the main products *via* the pathway involving SET, nickel catalysis, and energy transfer. The efficient isomerization for pure *E* product in the presence of [Ir(^*t*^Bu-ppy)_2_(dtbbpy)](PF_6_) also indicates the involvement of triplet–triplet energy transfer. Similarly, the scope with respect to the three components was broad, and the corresponding products could be delivered in good to high yields with good stereoselectivity.

**Scheme 15:**
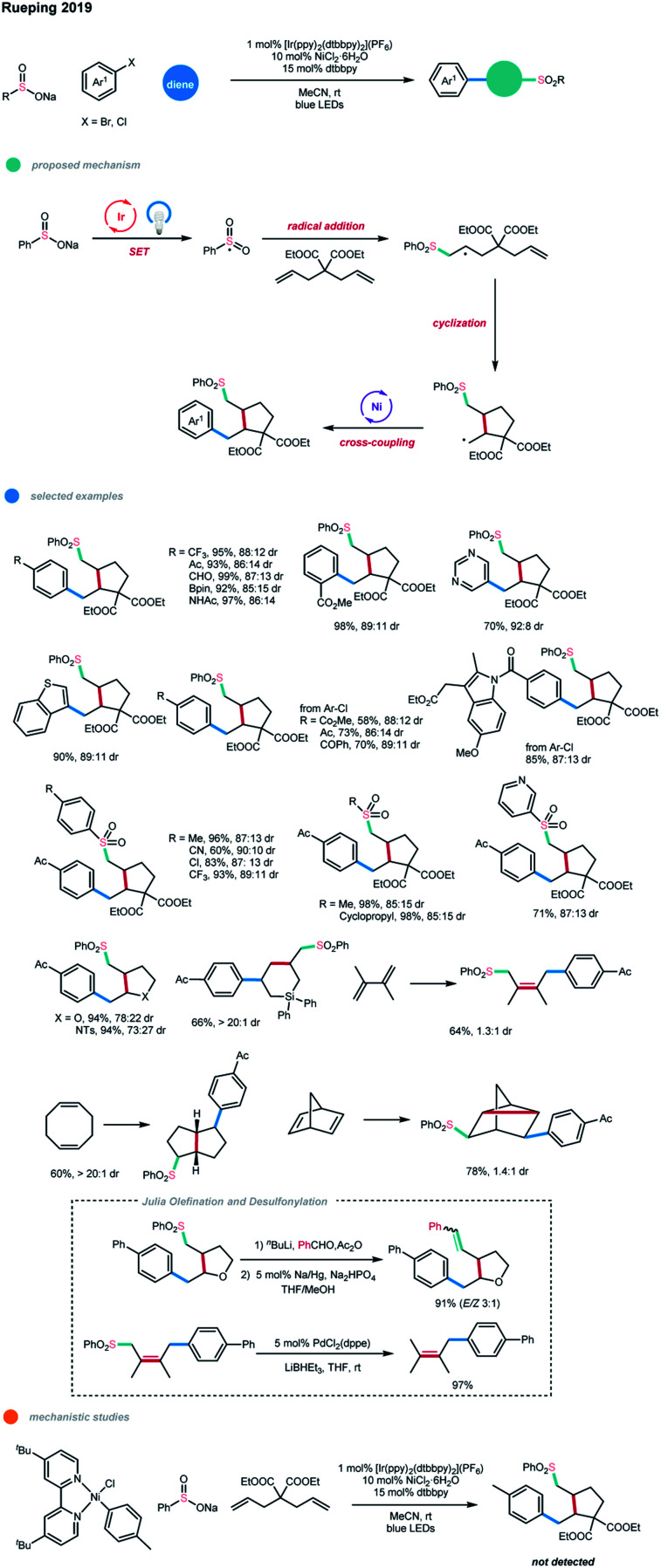
Cascade cross-coupling reactions of dienes *via* photoredox and nickel dual catalysis.

A short time later, based on the same concept, a cascade cross-coupling reaction of dienes *via* photoredox and nickel dual catalysis was realized by the same group (Scheme 15).^25^ In most cases, five-membered-ring products were obtained exclusively with the formation of two new C–C bonds and one C–S bond in one synthetic operation after the cyclization/cross-coupling of dienes with sodium sulfinates and (hetero)aryl halides. The sulfonyl radical is first generated from a SET oxidation step by an excited Ir photocatalyst. Subsequent radical addition and cascade cyclization give an alkyl radical species. The formed alkyl radical is then intercepted by the nickel catalyst and undergoes further cross-coupling with an aryl halide. The stoichiometric study with Ar–Ni^II^–Cl failed to give the cascade product, suggesting that it is not a competent intermediate, and the alternative catalytic Ni^0^/Ni^II^/Ni^III^ pathway is less favored. Under the optimized reaction conditions, a series of electronically unbiased 1,6-dienes, as well as heteroatom-containing dienes, could effectively undergo this reaction and give the corresponding five-membered-ring products in high yields with good diastereoselectivities.

Interestingly, for the allyldiphenyl silane substrate, only the corresponding six-membered ring product was observed *via* a 6-*endo*-trig cyclization, which may be due to factors such as the bond lengths, electronic effects, and conformation of the transition state. Moreover, polycyclic products could be synthesized from native cyclic 1,5- and 2,5-diene starting materials *via* this method, while 1,4-difunctionalization was observed for conjugated 1,3-dienes. Also, a wide range of electron-deficient, electron-neutral and electron-rich (hetero)aryl bromides and chlorides, including several pharmaceutically relevant aryl halides, all performed well in this cascade reaction. Additionally, both aryl and alkyl sodium sulfinates showed good reactivity. Notably, further Julia olefination and Pd-catalyzed desulfonylation of the generated sulfones were realized in excellent yields, highlighting the synthetic utility of this cascade method.

**Scheme 16:**
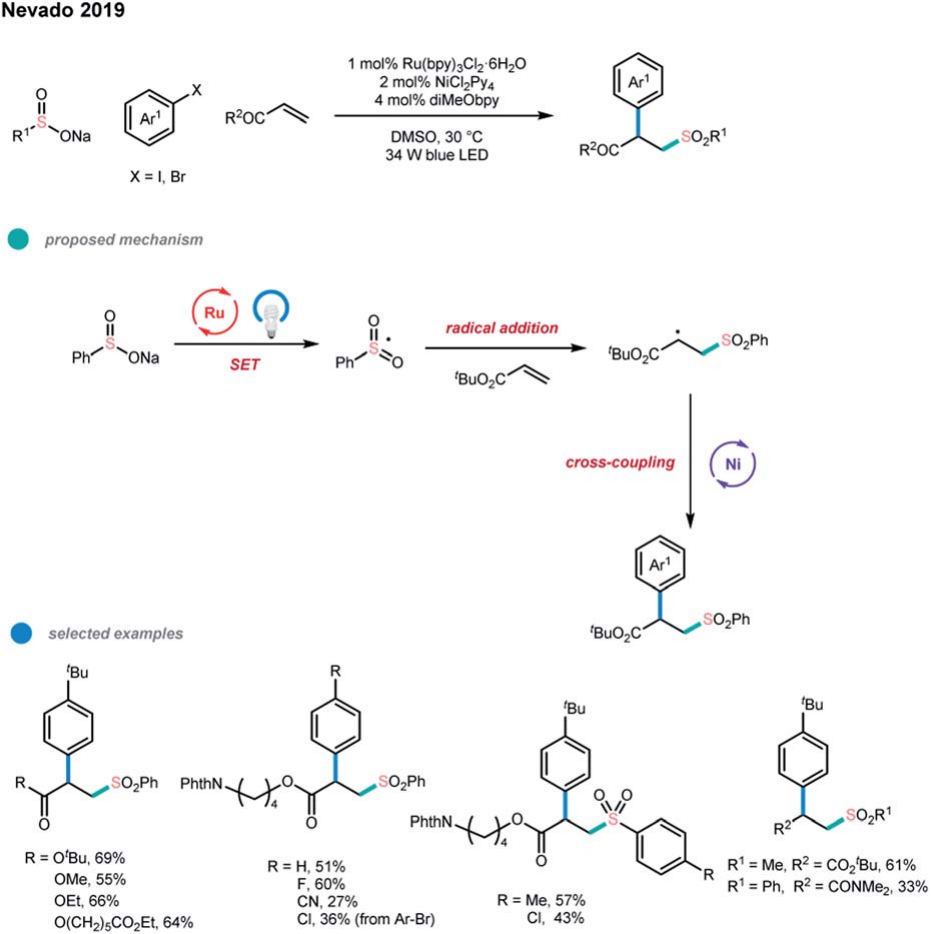
Three-component arylsulfonylation of alkenes *via* photoredox and nickel dual catalysis.

In the same year, Nevado and coworkers reported a three-component difunctionalization of alkenes (Scheme 16).^14^ In this paper, sulfonyl radicals were applied to realize the arylsulfonylation of alkenes *via* photoredox and nickel dual-catalyzed cascade reactions. Sulfonyl radicals generated from sodium sulfinate first added to the double bond and then underwent nickel-catalyzed cross-coupling reaction with the aryl halide. The optimized reaction conditions were as follows: 1 mol% Ru(bpy)_2_Cl_2_·6H_2_O as the photocatalyst, 2 mol% NiCl_2_(Py)_4_ as the nickel catalyst, and 4 mol% diMeObpy as the ligand in 0.1 M DMSO at 30 °C under irradiation with a 34 W blue LED.

Notably, lowering catalyst loading helps to suppress the formation of diarylsulfone byproduct, which is generated *via* the coupling of the sodium sulfinate and aryl iodide. A number of acrylates and aryl iodides are suitable substrates for this important transformation. Aryl bromides and vinyl amides could also be subjected to this catalytic system, although a higher catalyst loading (20 mol%) was needed.

## 5. Si-radical-involved photoredox and nickel dual-catalyzed cascade reactions

**Scheme 17:**
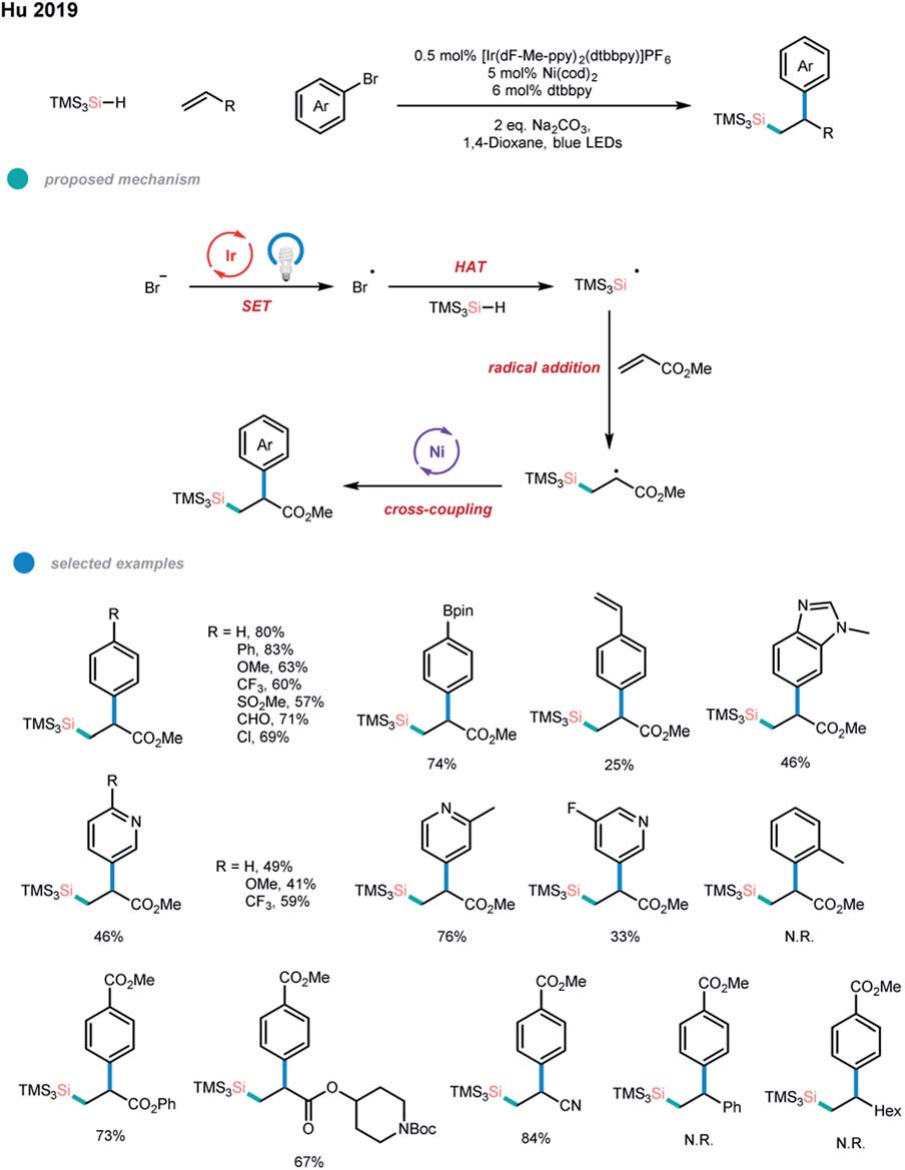
Arylsilylation of electron-deficient alkenes *via* photoredox and nickel dual catalysis.

Silyl radicals are often involved in the preparation of organosilicon compounds, and recently, a number of silylation reactions *via* photoredox catalysis under mild conditions have been developed.^26^ In particular, an arylsilylation of electron-deficient terminal alkenes *via* a photoredox and nickel dual-catalyzed cascade reaction was reported by Hu and coworkers (Scheme 17).^27^ In this protocol, an initial oxidation of Br^–^ by an excited photocatalyst gives Br˙, which undergoes subsequent hydrogen atom transfer (HAT) with TMS_3_SiH to form a silyl radical. The silyl radical is then trapped by an acrylate to generate a β-silyl alkyl radical, which then participates in the nickel catalytic cycle to deliver the final cascade coupling product.

A wide range of aryl and heteroaryl bromides could be applied in this arylsilylation cascade coupling, and diverse functional groups, including aldehyde, pinacolboronate ester, and bromide, were tolerated. Alkyl acrylates and acrylonitrile worked well and gave the corresponding products in good yields, while vinyl acetate gave low yield. Steric hindrance seems to substantially influence the efficiency of the three-component coupling since 1-bromonaphthalene, 2-methyl phenyl bromide and internal methyl acrylates gave no products, which may be due to the addition of an alkyl group with a large tri(trimethylsilyl)silyl group at the α-position to the nickel center being difficult. A limitation of this methodology is that it is so far not tolerant of styrenes, unactivated alkyl alkenes and other types of silane reagents.

## 6. Nonradical pathway cascade reaction

Notably, photoredox and nickel dual-catalyzed cascade coupling reactions involving non-radical pathways have also been reported.

**Scheme 18:**
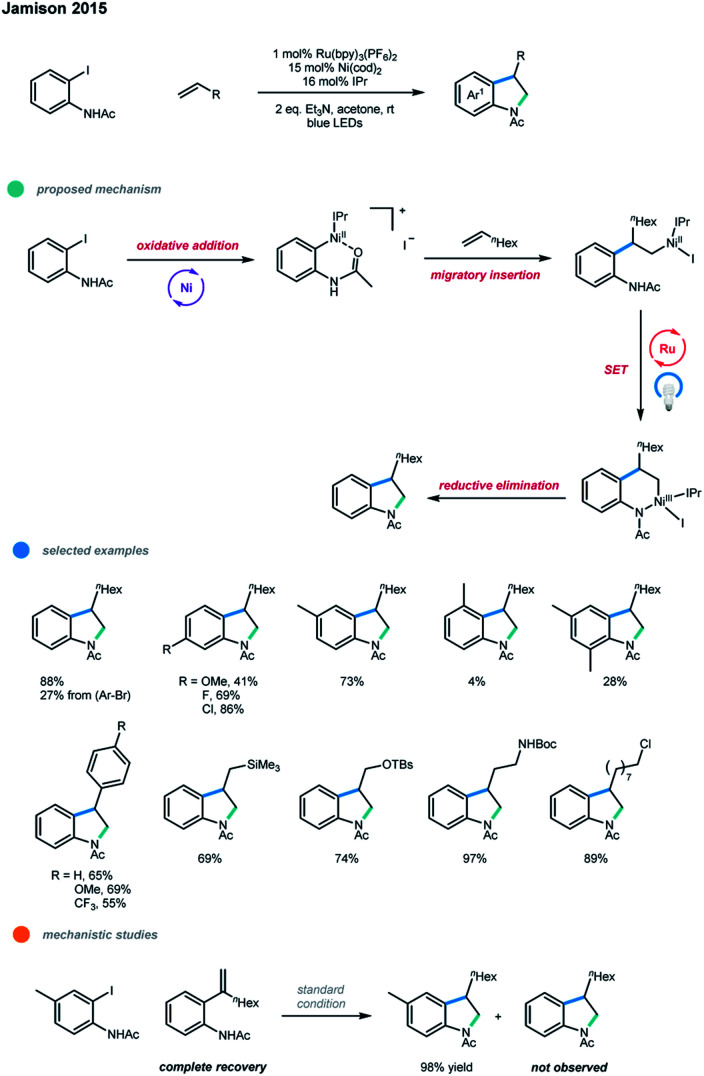
Regioselective indoline synthesis *via* photoredox and nickel dual catalysis.

In 2015, Jamison and coworkers developed a photoredox and nickel dual-catalyzed method to synthesize indolines in a highly regioselective manner (Scheme 18).^28^ One C(sp^2^)–C(sp^2^) bond and one C(sp^3^)–N bond were constructed in one synthetic operation in the cascade cross-coupling of iodoacetanilides and alkenes. A Ni^0/I/II/III^ mechanism was proposed.

First, oxidative addition of iodoacetanilide to Ni^0^ gives a Ni^II^ complex, which coordinates with an alkene and undergoes a migratory insertion to generate another Ni^II^ complex. This complex then undergoes ligand exchange with the pendent NH group, and subsequent oxidation by the excited Ru photocatalyst gives a Ni^III^ complex, which undergoes a reductive elimination to deliver the desired indoline product and a Ni^I^ complex. Ni^0^ is regenerated *via* a SET reduction by the photocatalyst. For the first time, the whole photoredox cycle involves direct modulation of the oxidation state of the nickel center, rather than oxidizing radical precursors to give radicals and reducing the nickel intermediates to those with lower oxidation states. In order to rule out another pathway including a discrete Heck reaction followed by hydroamination, the authors conducted the reaction of an iodoacetanilide with 1-octene in the presence of the possible Heck-type intermediate. The result shows that no product arising from the Heck-type intermediate was formed, indicating that this pathway is less favored. In addition, oxidants such as air and PhI(OAc)_2_ were helpful for the formation of the desired product when the reaction was conducted without photoredox catalyst, which suggests that Ni^III^ intermediate is likely involved before reductive elimination. In the optimized reaction conditions, the use of a Ni^0^ catalyst in the presence of the N-heterocyclic carbene (NHC) ligand IPr is critical to suppressing β-H elimination and highly selective alkene migratory insertion. A number of 4- and 5-substituted 2-iodoacetanilides work well in this protocol, while 3- or 6-substituted 2-iodoacetanilides gave dramatically lower yields. Aliphatic alkenes bearing trimethylsilyl groups, protected alcohols and amines, ketones, and alkyl chlorides, as well as electron-rich and electron-poor styrenes, were all suitable substrates, giving the corresponding products in good to excellent yields. However, internal and 1,1-disubstituted alkenes are unsuitable in this protocol, as they gave no desired products.

**Scheme 19:**
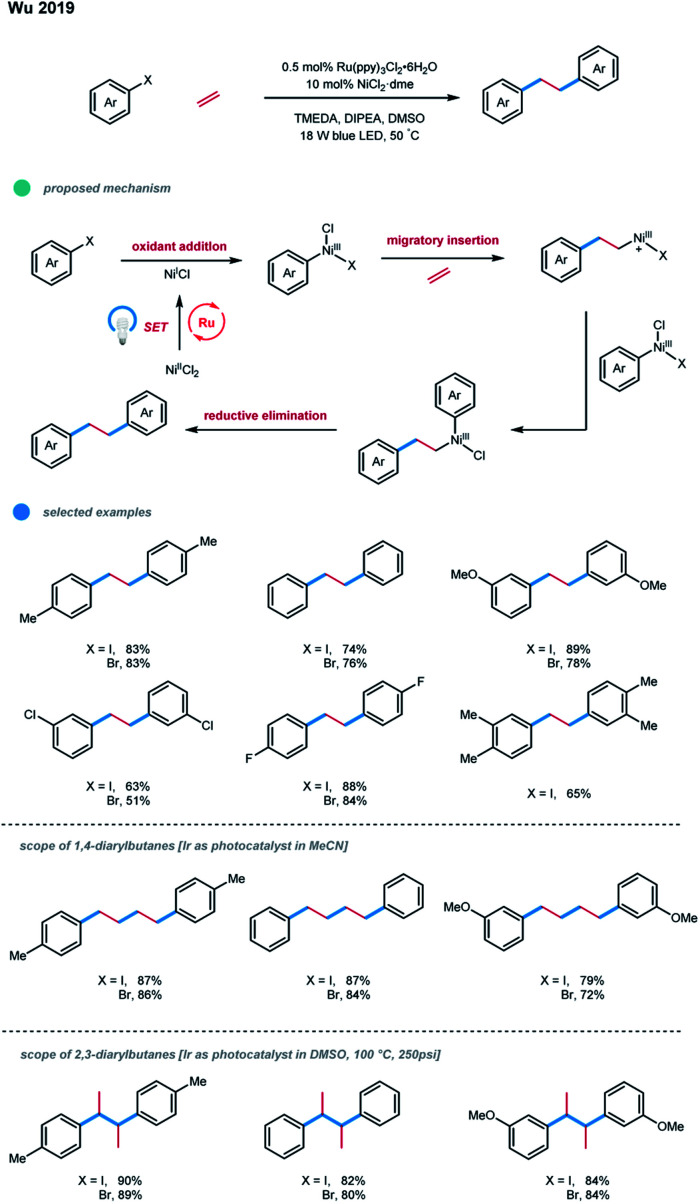
Ethylene diarylation *via* photoredox and nickel dual catalysis.

Recently, Wu and coworkers reported a photoredox/nickel catalyzed difunctionalization of ethylene with aryl halides in the presence of a stoichiometric amount of organic reductants, affording 1,2-diarylethanes with good efficiency (Scheme 19).^29^ In this process, the oxidative addition of an active catalyst Ni^I^ with an aryl halide would give an aryl–Ni^III^ species, which undergoes migratory insertion of ethylene to generate a cationic alkyl–Ni^III^ species with the dissociation of a halide ligand. Mechanistic studies and DFT calculations provided support for this proposed pathway. Subsequent aryl transfer would deliver the crucial (alkyl)(aryl)–Ni^III^ species, which can undergo a facile reductive elimination to furnish the desired diarylation product. A series of electron-rich and electron-neutral aryl iodides and bromides worked well, generating the linear diarylethanes from ethylene feedstock with good to high efficiency. Nevertheless, aryl halides with *ortho*-substituents or electron-deficient substituents were not suitable substrates, probably due to steric hindrances or the competitive dehalogenation in the presence of a reductant.

Interestingly, using photocatalysts with proper redox potentials and tuning the reaction parameters allowed 1,4-diarylbutanes and 2,3-diarylbutanes to be formed selectively. Although the selectivity is not fully understood, the authors found that the Ru-catalyst favored the 1,2-diarylethane products, the Ir-catalyst tended to give 1,4-diarylbutane products, and temperature higher than 100 °C led to the high selectivity of 2,3-diarylbutane products.

## 7. Conclusions

In this review, we have highlighted the recent progress in photoredox and nickel-catalyzed cascade reactions and presented the classification of the cascades useful for carbon–carbon and carbon heteroatom bound formations based on the initially formed C-, N-, S-, and Si- radicals. The illustrated methodologies have gained considerable interest in the past years as useful tool for the efficient construction of complex molecular structures from readily available starting materials. Due to the mild reaction conditions, most of the protocols presented here show high efficiency and stereo- and regioselectivity, good functional group tolerance and good scalability. Additional benefits of photoredox/metal dual cascade reactions include high atom-economy, reduced waste generation and high synthetic efficiency. Although the field of photoredox mediated cascade reactions is relatively new, it has experienced a rapid and astonishing progress in a short period of time. The extension of the successfully employed radicals to more diverse cascade reactions and the application of other types of radicals such as O-, P- radicals are expected to be developed. Since often multiple stereocenters are generated the photoredox and nickel catalysed cascade reaction platform also provides a great opportunity to develop enantioselective variants.

## Conflicts of interest

There are no conflicts to declare.
